# The burden of atherosclerotic cardiovascular disease among working-age people across SDI regions: an age–period–cohort study based on the GBD 2021

**DOI:** 10.3389/fcvm.2025.1609672

**Published:** 2025-09-02

**Authors:** Yuan Yan, Jing Xie, Hui Wu, Yong-Gang Tang, Yang-Guang Yin

**Affiliations:** ^1^ Center for Global Health, School of Public Health, Nanjing Medical University, Nanjing, China; ^2^ Department of Intensive Care Unit, The Second Hospital of Nanjing, Affiliated to Nanjing University of Chinese Medicine, Nanjing, China; ^3^ Chongqing Prevention and Treatment Center for Occupational Diseases, The First Affiliated Hospital of Chongqing Medical and Pharmaceutical College, Chongqing, China

**Keywords:** atherosclerotic cardiovascular disease, working-age population, age-period-cohort analysis, global burden of disease, socio-demographic index, ischemic heart disease, ischemic stroke, peripheral artery disease

## Abstract

**Background:**

Atherosclerotic cardiovascular disease (ASCVD) remains a leading health threat to the working-age population globally, with marked disparities in incidence and mortality across countries at different levels of socio-demographic development.

**Methods:**

Based on data from the Global Burden of Disease Study 2021, we used an age-period-cohort (APC) model to evaluate trends in the incidence and mortality of three major ASCVD subtypes—ischemic heart disease (IHD), ischemic stroke (IS), and peripheral artery disease (PAD)—among working-age adults (15–64 years) from 1992 to 2021 across five socio-demographic index (SDI) regions. The model further explored the contributions of age, period, and cohort effects.

**Results:**

Although age-standardized incidence and mortality rates of ASCVD have declined in most high-SDI regions and globally, the absolute number of cases and deaths has continued to rise, especially in middle- and low-SDI regions. Notably, PAD showed increasing incidence trends in several developing regions, suggesting persistent gaps in public awareness and preventive efforts.

**Conclusion:**

ASCVD remains a significant global public health challenge among the working-age population. SDI-specific and accessible interventions, along with strengthened cross-sector collaboration, are urgently needed to promote effective prevention and control of ASCVD worldwide.

## Introduction

As a major contributor to the global burden of disease and mortality, cardiovascular diseases (CVDs) remain a critical global health concern (1). Driven by a rising global burden, the incidence of CVDs has nearly doubled over the past three decades. In 2019, these conditions led to around 18.6 million deaths, constituting nearly one-third of all deaths worldwide (2). Atherosclerotic cardiovascular disease (ASCVD), the predominant pathological subtype of CVD, continues to constitute the major contributor to CVD-related morbidity and mortality and plays a significant role in global health disparities (3).

ASCVD, comprising ischemic heart disease (IHD), ischemic stroke (IS), and peripheral artery disease (PAD), is the most common form of cardiovascular disease (4). Among CVD-related deaths, IHD accounts for 49.2% and IS for 17.7%, making them the leading causes of CVD mortality worldwide (2). While recent global assessments have reported the burden and trends of CVD between 1990 and 2021, the specific global burden of ASCVD has not been comprehensively documented.

The working-age population, or labor force, is typically defined as individuals aged 15–64 years worldwide (5). Currently, the rising incidence and mortality rates of ASCVD among this group pose a serious threat to national prosperity, family stability, and societal well-being, resulting in a substantial social and economic burden. Evidence has shown that long working hours, job-related stress, and occupational strain are associated with an increased risk of ASCVD among working-age individuals (6–8). To date, most estimates of the CVD burden have focused on the total population at the global, regional, and national levels (2), with limited attention paid to the burden of ASCVD among working-age populations—who are key contributors to both households and society at large.

To fill this gap, we estimated the incidence and mortality of ASCVD to systematically assess the disease burden among working-age subpopulations stratified by sociodemographic index (SDI). Based on GBD 2021 data, an age–period–cohort (APC) model was employed to capture temporal dynamics in ASCVD incidence and mortality among the working-age population globally and across SDI regions from 1990 to 2021.

## Materials and methods

### Data source

Health burden estimates were derived from the Global Burden of Disease 2021 dataset, a comprehensive initiative led by the Institute for Health Metrics and Evaluation (IHME) to generate comparable data on diseases and injuries worldwide. Publicly accessible via the Global Health Data Exchange (GHDx), the GBD 2021 dataset offers estimates for 204 countries and territories, including subnational breakdowns for 21 of them, covering the period from 1990 to 2021. Metrics span 371 diseases and injuries, including mortality, incidence, prevalence, and key composite indicators of disease burden and healthy life expectancy (3, 9, 10). This rich dataset serves as one of the most comprehensive resources for assessing disease burden, risk factors, mortality, and disability worldwide. Furthermore, GBD 2021 incorporates analyses related to COVID-19, including estimates of the burden of long COVID, excess mortality caused by the pandemic, and adjustments to the prevalence of depressive and anxiety disorders reflecting the pandemic's mental health impacts (11).

### Estimation of ASCVD burden in the working-age population

Given that the primary subtypes of ASCVD include IHD, IS, and (PAD, our study incorporated annual data on these three ASCVD subtypes for analysis. In this study, we defined the working-age population as individuals aged 15–64 years according to standard demographic classifications. However, peripheral arterial disease (PAD) data provided by the Global Burden of Disease (GBD) 2021 database are only available for individuals aged 40 years and older. Therefore, for analytical consistency, PAD incidence and burden in the 15–39-year age group were assumed to be zero, as no data were available. This assumption was made solely to maintain structural compatibility with the age-period-cohort (APC) model and does not imply a true absence of disease in that population. Based on the data range of the GBD 2021 database and the definition of the working-age population (5), we included individuals aged 15–64 years. Age-, sex-, and region-specific incidence and mortality estimates for IHD, IS, and PAD were derived from the GBD 2021 Study. The data are publicly available via the GBD Results Tool. Detailed information on the modeling strategies used in GBD 2021 has been published in previous studies (3, 9, 10).

### Socio-demographic index

As a summary index, SDI quantifies the socio-demographic advancement of nations or regions. The index ranges between 0 and 1, with increasing values denoting higher socio-economic advancement (12). Previous studies have shown significant associations between SDI and both the incidence and mortality of various diseases. Based on SDI values in GBD 2021, countries were grouped into five development levels: low (<0.47), low-middle (0.47–0.62), middle (0.62–0.71), high-middle (0.71–0.81), and high (>0.81) (13). We therefore investigated how ASCVD burden relates to socio-economic development across five SDI-defined groups of countries and regions, from low to high development levels.

### Statistical analysis

This study used an APC modeling approach to quantify the respective impacts of age, period, and cohort dimensions on time-based differences in ASCVD incidence and mortality, thereby providing insights into temporal dynamics and potential drivers of disease burden. The APC model yields several key indicators: net and local drifts, age-specific trends, and period- and cohort-specific relative risks (14). Net drift estimates the general annual rate of change in incidence or mortality, whereas local drift identifies age-specific patterns within the time dimension. The longitudinal age curve depicts age-specific estimates for the reference cohort after adjustment for period influences. Period and cohort relative risks represent the risk in each time segment relative to a defined reference group; RRs > 1 indicate increased ASCVD risk, whereas RRs < 1 suggest a reduced risk. The significance of model estimates and related functions was assessed using Wald tests, and all statistical analyses were performed using two-sided tests. To meet the structural requirements of the APC model, we divided both age and period into equal 5-year intervals. Although GBD 2021 provides data starting from 1990, we excluded the years 1990 and 1991 because they do not form a complete 5-year period. According to APC modeling guidelines, the age and period intervals must be of equal length to ensure valid decomposition of temporal effects (15). Therefore, our analysis began in 1992, which marks the start of the first full 5-year interval (1992–1996). We then adopted the modeling procedures recommended by the APC Web Tool (U.S. National Cancer Institute), with technical documentation publicly accessible online (15). All analyses were conducted in R (4.4.2), using *easyGBD* for computation and *ggplot2* for plotting.

## Results

Table 1 shows the absolute numbers of ASCVD deaths and incident cases, corresponding mortality and incidence rates (both all-age and age-standardized), and net drift values across global and SDI-defined regions. Over the last 30 years, an upward trend in mortality due to IHD, IS, and PAD has been documented worldwide, with respective increases of 41.93%, 24.04%, and 36.75%. Most SDI regions exhibited comparable patterns, with ASCVD-related deaths increasing notably across middle- to low-SDI groups. By contrast, the high-middle SDI region experienced a decrease. For IHD and IS, the net drift values of mortality showed a declining trend globally and across most regions, indicating an overall annual reduction in mortality rates. However, for PAD, negative net drift values were observed only at the global level and in the high-middle SDI region, while all other regions had positive values, suggesting a year-on-year increase in PAD mortality rates elsewhere.

**Table 1:** Trends in incidence and mortality of ischemic heart disease, ischemic stroke, and peripheral artery disease worldwide and across five SDI regions, 1992–2021.

Disease/indicator				Global		SDI									
						High SDI		High middle		Middle SDI		Low middle		Low SDI	
				1992	2021	1992	2021	1992	2021	1992	2021	1992	2021	1992	2021
IHD	Death		Number,*n* × 1,000	1,601.75 (1,541.62, 1,663.61)	2,273.32 (2,140.13, 2,413.46)	283.40 (278.03, 288.16)	201.43 (192.85, 210.76)	438.07 (423.34, 453.80)	413.93 (379.10, 453.38)	421.50 (397.90, 445.79)	773.69 (720.79, 833.27)	352.62 (324.51, 381.57)	690.56 (629.40, 753.31)	103.80 (90.33, 118.36)	191.66 (169.59, 216.14)
			Percent change in deaths from 1992 to 2021,%	41.93%		−28.93%		−5.51%		83.56%		95.84%		84.64%	
			All-age mortality rate per 100,000	47.39	44.49	47.37	28.08	60.48	46.54	37.95	46.85	52.29	56.29	38.54	30.91
			Age-standardized mortality rate per 100,000	55.87	41.69	44.06	20.27	62.57	35.03	49.87	42.41	70.15	65.77	56.22	47.35
			Percentage change in mortality rate from 1992 to 2021,%	−25.38%		−53.99%		−44.01%		−14.96%		−6.24%		−15.78%	
		APC model estimates	Net drift in mortality rate,% per year	−0.86 (−0.93, −0.80)		−2.01 (−2.16, −1.87)		−2.30 (−2.39, −2.21)		−0.47 (−0.54, −0.39)		−0.35 (−0.44, −0.26)		−0.70 (−0.82, −0.57)	
	Incidence		Number, *n* × 1,000	7,332.60 (4,863.16, 10,471.32)	13,740.02 (8,939.32, 19,923.32)	1,280.40 (868.99, 1,813.82)	1,317.99 (858.49, 1,901.81)	1,715.86 (1,148.83, 2,445.32)	2,817.48 (1,804.86, 4,132.61	1,943.04 (1,241.57, 2,837.72)	4,592.60 (2,941.32, 6,726.66)	1,810.42 (1,218.33, 2,570.36)	3,794.98 (2,543.22, 5,391.79)	575.32 (374.29, 829.99)	1,206.92 (765.77, 1,764.76)
			Percent change in incidence cases from 1992 to 2021,%	87.38%		2.94%		64.20%		136.36%		109.62%		109.69%	
			All-age incidence rate per 100,000	216.93	268.92	214.02	183.72	236.88	316.81	174.95	278.08	268.45	309.34	213.63	194.62
			Age-standardized incidence rate per 100,000	256.04	251.61	200.56	137.5	245.94	241.39	230.51	250.09	365.19	362.11	310.63	295.3
			Percentage change in incidence rate from 1992 to 2021,%	−1.73%		−31.44%		−1.85%		8.49%		−0.84%		−4.94%	
		APC model estimates	Net drift in incidence rate,% per year	0.14 (0.07, 0.22)		−0.79 (−0.91, −0.67)		−0.02 (−0.15, 0.10)		0.38 (0.29, 0.47)		0.31 (0.22, 0.41)		0.02 (−0.04, 0.08)	
IS	Death		Number,*n* × 1,000	310.59 (287.47, 339.77)	385.27 (348.89, 427.36)	33.25 (31.84, 34.72)	22.61 (20.73, 24.85)	116.53 (109.48, 124.51)	100.60 (89.28, 113.27)	92.24 (82.67, 104.88)	141.65 (125.40, 160.38)	50.81 (42.96, 61.05)	90.19 (77.21, 109.26)	17.37 (13.67, 23.63)	29.88 (23.87, 40.15)
			Percent change in deaths from 1992 to 2021,%	24.04%		−32.00%		−13.67%		53.57%		77.50%		72.02%	
			All-age mortality rate per 100,000	9.19	7.54	5.56	3.15	16.09	11.31	8.31	8.58	7.53	7.35	6.45	4.82
			Age-standardized mortality rate per 100,000	10.99	7.03	5.11	2.21	16.4	8.29	11.41	7.78	10.62	8.89	9.86	7.93
			Percentage change in mortality rate from 1992 to 2021,%	−36.03%		−56.75%		−49.45%		−31.81%		−16.29%		−19.57%	
		APC model estimates	Net drift in mortality rate,% per year	−1.37 (−1.44, −1.29)		−2.00 (−2.19, −1.80)		−2.48 (−2.61, −2.35)		−1.20 (−1.34, −1.06)		−0.63 (−0.72, −0.55)		−0.60 (−0.74, −0.47)	
	Incidence		Number, *n* × 1,000	1,667.57 (1,129.41, 2,352.29)	2,714.32 (1,897.96, 3,763.26)	347.68 (242.34, 481.51)	366.52 (258.15, 504.03)	490.94 (337.57, 685.75)	664.30 (469.42, 918.11)	454.56 (299.16, 653.41)	944.13 (659.39, 1,311.44)	260.81 (173.02, 373.95)	517.05 (356.54, 719.02)	111.65 (74.50, 160.09)	220.18 (149.33, 310.19)
			Percent change in incidence cases from 1992 to 2021,%	62.77%		5.42%		35.31%		107.70%		98.25%		97.21%	
			All-age incidence rate per 100,000	49.33	53.13	58.11	51.09	67.77	74.7	40.93	57.17	38.67	42.15	41.46	35.5
			Age-standardized incidence rate per 100,000	57.11	50.13	54.72	39.14	70.05	57.73	52.14	52.07	49.65	47.76	55.99	48.96
			Percentage change in incidence rate from 1992 to 2021,%	−12.22%		−28.47%		−17.59%		−0.13%		−3.81%		−12.56%	
		APC model estimates	Net drift in incidence rate,% per year	−0.33 (−0.36, −0.30)		−0.85 (−0.89, −0.81)		−0.64 (−0.69, −0.58)		−0.07 (−0.12, −0.03)		−0.002 (−0.049, 0.045)		−0.40 (−0.43, −0.38)	
PAD	Death		Number,*n* × 1,000	5.47 (5.05, 5.99)	7.48 (6.76, 8.69)	1.61 (1.56, 1.67)	2.33 (2.21, 2.49)	2.85 (2.66, 3.14)	2.40 (2.17, 2.74)	0.58 (0.52, 0.63)	1.41 (1.25, 1.59)	0.24 (0.16, 0.36)	0.79 (0.62, 1.08)	0.18 (0.09, 0.36)	0.52 (0.28, 1.00)
			Percent change in deaths from 1992 to 2021,%	36.75%		44.72%		−15.79%		143.10%		229.17%		188.89%	
			All-age mortality rate per 100,000	0.48	0.35	0.64	0.64	1.07	0.53	0.17	0.1945	0.12	0.19	0.24	0.31
			Age-standardized mortality rate per 100,000	0.5	0.34	0.63	0.56	1.02	0.49	0.18	0.1941	0.13	0.2	0.26	0.36
			Percentage change in mortality rate from 1992 to 2021,%	−32.00%		−11.11%		−51.96%		7.83%		53.85%		38.46%	
		APC model estimates	Net drift in mortality rate,% per year	−1.79 (−1.96, −1.62)		−0.87 (−1.20, −0.53)		−3.56 (−3.84, −3.27)		−0.26 (−0.64, 0.12)		1.52 (0.91, 2.13)		0.97 (0.23, 1.72)	
	Incidence		Number, *n* × 1,000	2,365.33 (1,693.84, 3,157.84)	4,297.08 (3,070.57, 5,742.50)	778.34 (561.65, 1,041.78)	1,003.06 (732.45, 1,323.49)	591.64 (422.27, 790.04)	1,030.61 (730.44, 1,386.34)	621.28 (441.02, 835.81)	1,418.32 (1,002.08, 1,919.95)	284.48 (202.05, 385.09)	640.33 (454.35, 868.27)	87.39 (62.13, 118.49)	201.49 (143.11, 273.52)
			Percent change in incidence cases from 1992 to 2021,%	81.67%		28.87%		74.20%		128.29%		125.09%		130.56%	
			All-age incidence rate per 100,000	209.69	201.32	309.87	275.44	223.29	229.5	185.66	195.88	141.63	150.91	116.24	117.77
			Age-standardized incidence rate per 100,000	213.26	198.59	307.02	253.6	218.58	218.41	193.6	193.76	148.94	157.18	123.28	130.26
			Percentage change in incidence rate from 1992 to 2021,%	−6.88%		−17.40%		−0.08%		0.08%		5.53%		5.66%	
		APC model estimates	Net drift in incidence rate,% per year	−0.58 (−9.39, 9.09)		−0.60 (−22.27, 27.11)		0.07 (−5.11, 5.54)		−0.25 (−5.41, 5.20)		−0.54 (−5.69, 4.90)		−0.89 (−6.02, 4.53)	

All-age incidence rate was defined as the crude incidence rate.

All-age mortality rate was defined as the crude mortality rate.

Age-standardized incidence and mortality rates were calculated by direct standardization using the global standard population in GBD 2021.

Net drift was estimated by the age–period–cohort (APC) model, representing the overall annual percentage change in incidence and mortality rates, which reflects the combined effects of calendar period (period effect) and successive birth cohorts (cohort effect).

Parentheses for net drift indicate 95% confidence intervals.

APC, age–period–cohort; GBD, Global Burden of Disease.

From 1992 to 2021, the overall incidence trend of ASCVD showed a general decline, although the absolute number of new cases increased. For IHD, negative net drift values in incidence were observed globally and across non-middle SDI regions, reflecting a downward trend, while the middle SDI region exhibited a positive value. For IS, net drift values indicated a consistent downward trend globally and among all SDI groups. In PAD, apart from the high-middle SDI group, all other regions showed negative net drift values, indicating a general decline in incidence rates over time.

### APC effects on ASCVD incidence and mortality in working-age population

**Figure 1:**
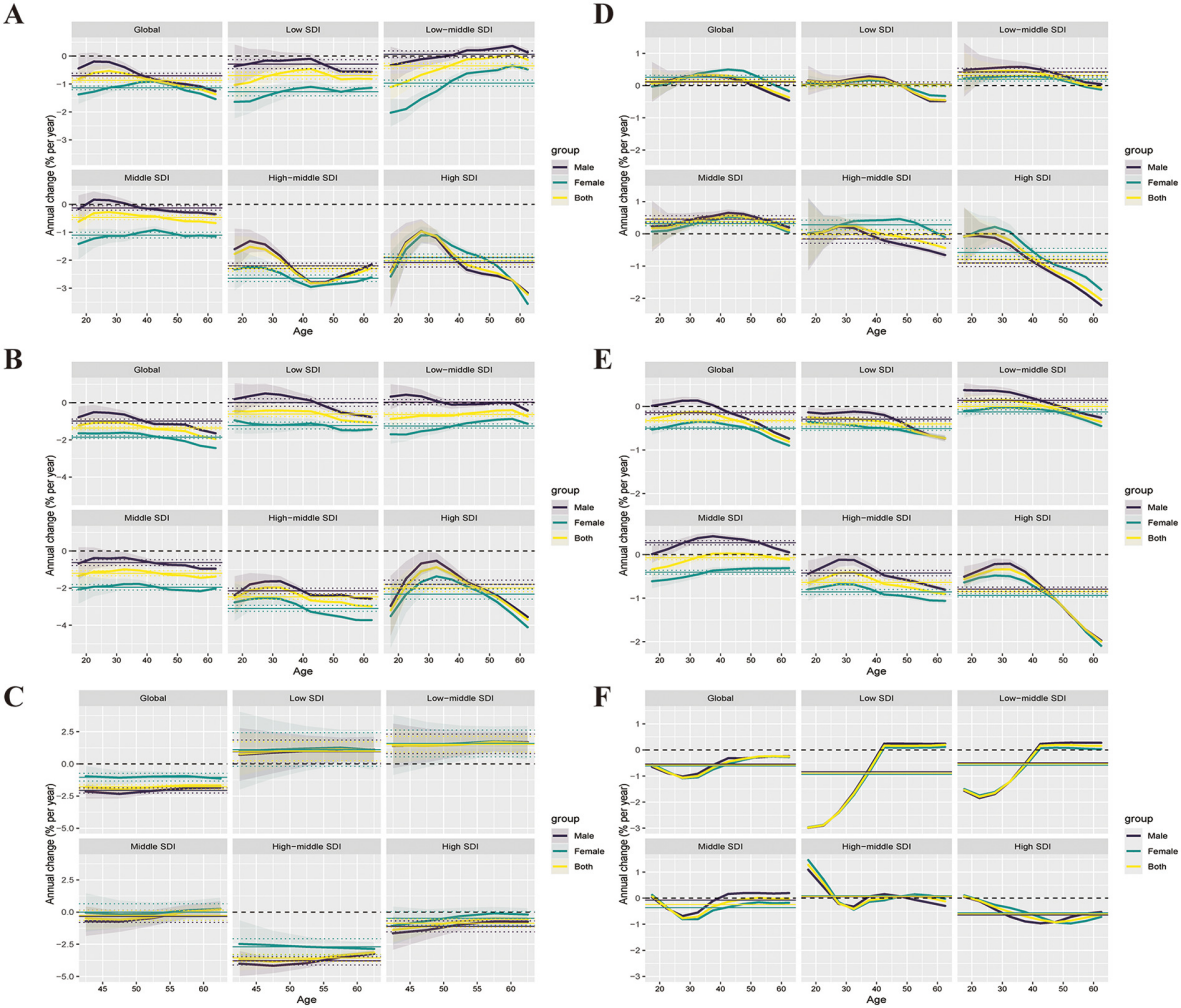
Effects of ASCVD on mortality and incidence among the working-age population globally and across the five SDI regions from 1992 to 2021. **(A–C)** show the local drifts in mortality rates for IHD, IS, and PAD, respectively, across ten age groups (15–64 years), as estimated by the age–period–cohort model. **(D–F)** display the local drifts in incidence rates for IHD, IS, and PAD across the same age groups. Dots represent the estimated annual percentage change (% per year), and shaded areas indicate the corresponding 95% confidence intervals (95% CIs).

Figure 1 illustrates the annual percentage change in mortality and incidence rates of IHD, IS, and PAD across 5-year age groups among individuals aged 15–64.

Figure 1A displays the age-specific local drift values for IHD mortality. Globally, most age groups showed negative local drift, indicating an overall decline in IHD mortality. A similar trend was observed in low SDI regions. However, in high to middle SDI regions, individuals under the age of 34 showed positive local drift, suggesting increased mortality in younger age groups. Conversely, within the low-middle SDI group, a different pattern emerged, with positive local drift appearing in individuals aged over 40, suggesting elevated mortality risk with advancing age.

Figure 1B depicts the time trends of IS mortality, expressed as annual percentage change. Globally, IS mortality exhibited a predominantly negative local drift across age groups, suggesting a downward trend. In countries and regions with low and low-middle SDI, however, positive local drift was observed in those under 45 and 30 years of age, respectively, indicating an increasing mortality trend among younger individuals. Other regions followed the global trend with mostly negative values.

Figure 1C presents the annual percentage change in PAD mortality among individuals aged 40–64. Globally, PAD mortality showed predominantly negative local drift, suggesting a general decline. Conversely, in low and low-middle SDI groups, an overall rise in mortality was indicated by positive local drift values in nearly all age groups. Similarly, most age groups in middle SDI regions showed increasing trends. Conversely, negative local drift in high and high-middle SDI groups reflected ongoing reductions in mortality.

Figure 1D illustrates the local drift of IHD incidence. Globally, individuals aged 25–55 showed positive local drift values, suggesting increasing incidence in these age groups. In middle and low SDI groups, individuals younger than 55 years also showed positive local drift, indicating an upward trend in IHD incidence. Figure 1E presents the annual percentage change in IS incidence. Globally and in low-middle SDI regions, positive local drift values were observed in several age groups, indicating increasing incidence. In comparison, low, high, and high-middle SDI regions exhibited predominantly negative local drift, suggesting a declining incidence trend. Notably, nearly all age groups in the middle SDI region exhibited positive local drift values, indicating a widespread increase in IS incidence.

Figure 1F illustrates the age-specific patterns of PAD incidence, with negative local drift observed in the majority of age groups worldwide—suggesting an overall downward trend. Conversely, in low and low-middle SDI groups, individuals aged over 40 exhibited positive local drift values, indicating increased PAD incidence among older working-age populations.

### APC effects on ASCVD in the working-age population

#### IHD

**Figure 2:**
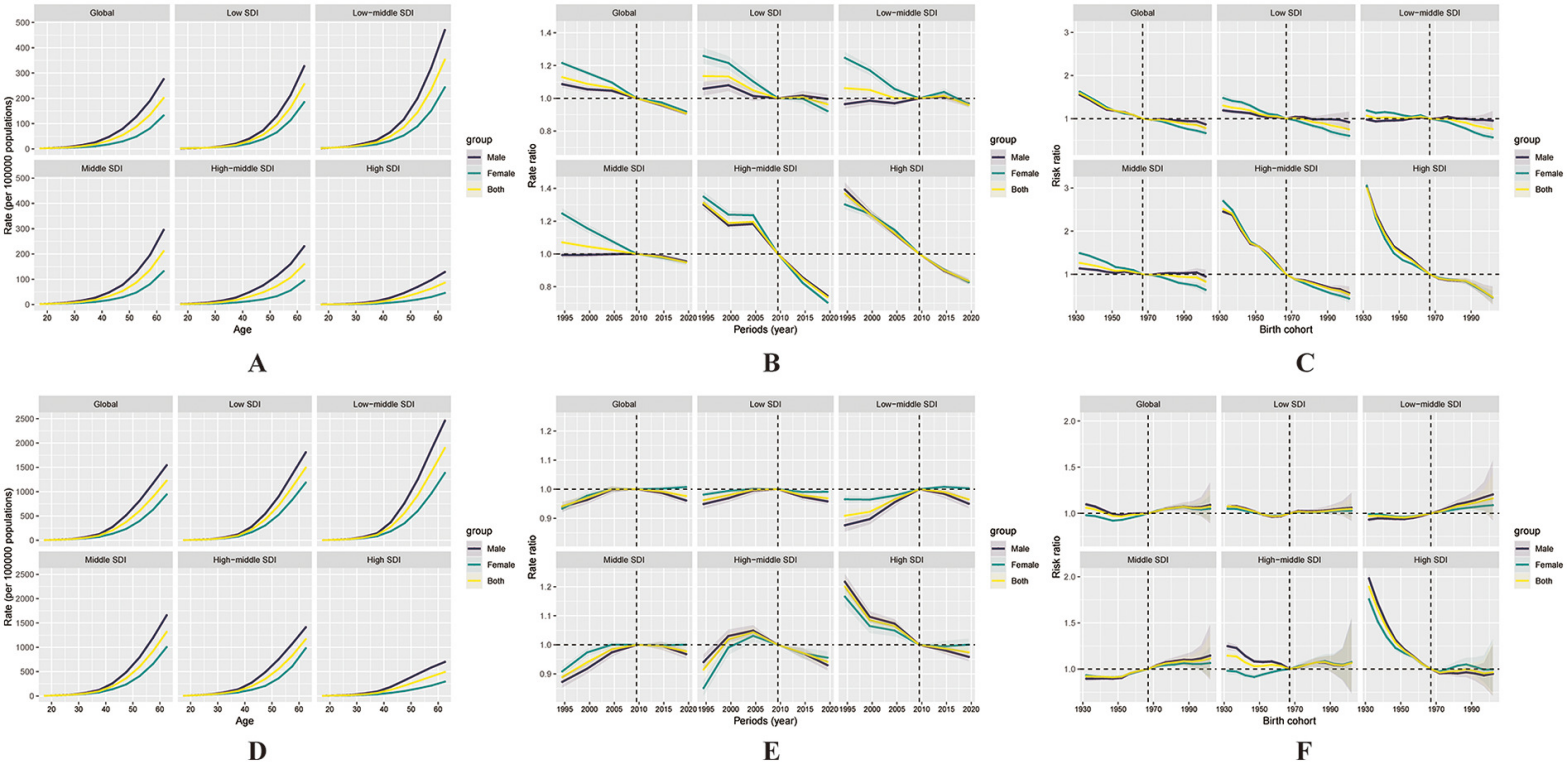
Illustrates the effects of age, period, and cohort on ischaemic heart disease (IHD) mortality and incidence among the working-age population globally and across the five SDI regions. Age effects on mortality **(A)** and incidence **(D)** are presented as longitudinally fitted age-specific rates, adjusted for period deviations. Period effects **(B, E)** are shown as relative risks of mortality and incidence, with 2007 set as the reference year. Cohort effects **(C, F)** are expressed as relative risks for mortality and incidence, respectively. In all panels, the dots represent point estimates, and the shaded areas indicate the corresponding 95% confidence intervals.

Figures 2A–C illustrates the estimated APC effects on ASCVD mortality at the worldwide level and across the five SDI regions. Mortality risk increased with age in a consistent pattern observed across all countries and regions. This age-related increase was particularly pronounced in the low SDI region. On a global scale, the period effect remained relatively stable, while sustained declines were observed across high to middle SDI regions, indicating effective control of mortality over time. Conversely, countries in the low to low-middle SDI categories demonstrated periods of elevated risk above the reference level, suggesting a less favorable trend in mortality control. Cohort effects appeared relatively stable over time, both globally and across all SDI regions.

Figures 2D–F presents the estimated APC effects on the incidence of IHD. The age effect showed that incidence risk increased with age globally and across all SDI regions. Notably, the increase was more gradual in the high SDI region, indicating better control of IHD incidence in that setting. Period effects remained relatively unchanged over the past three decades at the global level, with consistent trends also noted in middle- and lower-SDI regions. Of particular interest, the high-middle and high SDI region exhibited a positive period effect beginning around 2001, followed by a subsequent decline. In the high SDI region, the period effect transitioned from positive to negative values, reflecting a decreasing trend in incidence. Regarding cohort effects, a gradual increase in incidence risk across successive birth cohorts was observed globally and across low to middle SDI regions over the past three decades. Conversely, high-middle and high SDI groups exhibited a downward cohort trend, indicating lower incidence risks among more recent cohorts.

#### IS

**Figure 3:**
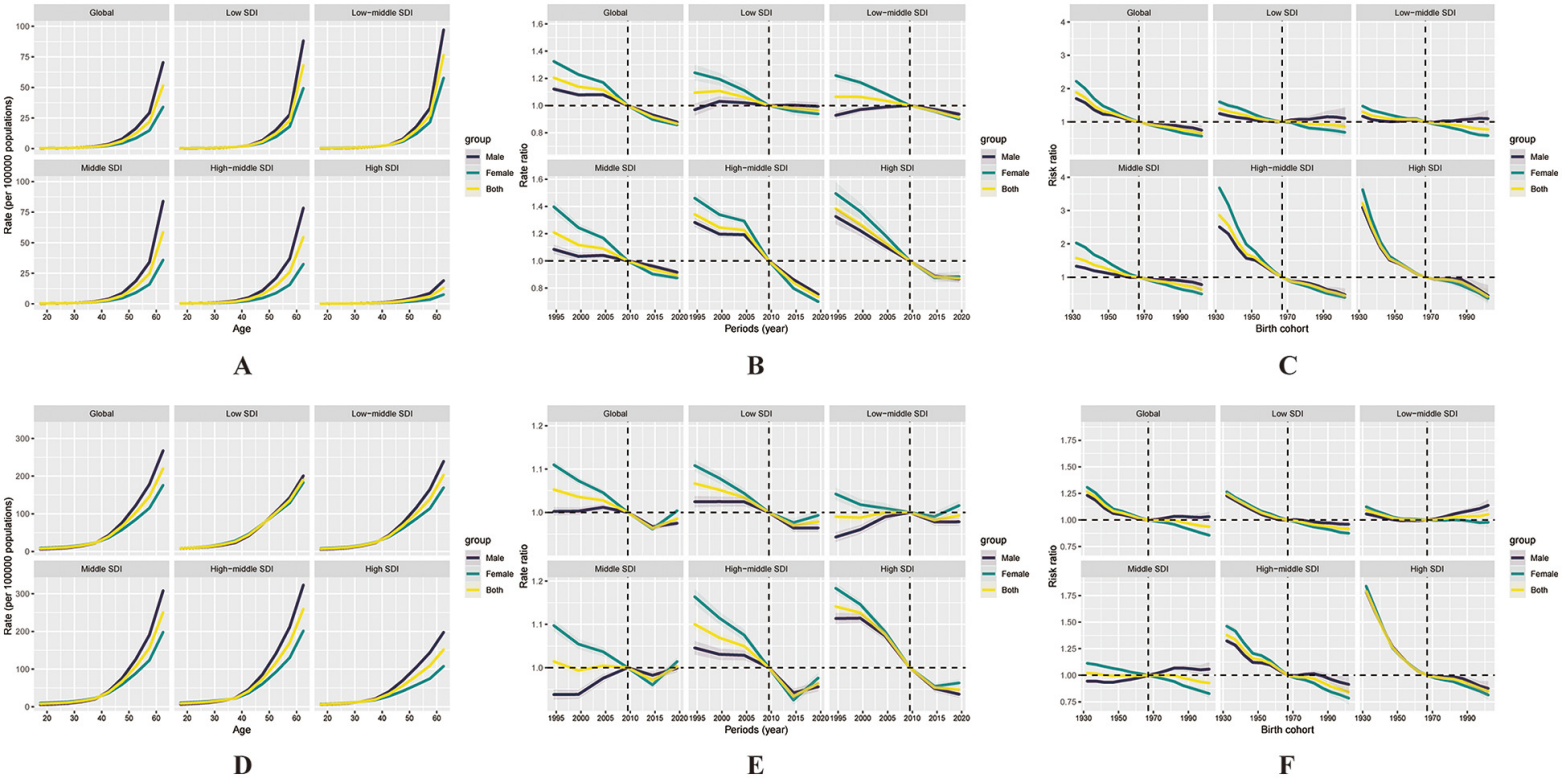
Illustrates the effects of age, period, and cohort on ischaemic stroke (IS) mortality and incidence among the working-age population globally and across the five SDI regions. Age effects on mortality **(A)** and incidence **(D)** are presented as longitudinally fitted age-specific rates (per 100,000 person-years), adjusted for period deviations. Period effects **(B, E)** are shown as relative risks of mortality and incidence, respectively, with 2007 designated as the reference year. Cohort effects **(C, F)** are expressed as relative risks for mortality and incidence. In all panels, the dots represent point estimates of the rates or relative risks, and the shaded areas indicate the corresponding 95% confidence intervals.

Figures 3A–C presents the APC model estimates for IS mortality. Globally, the age effect followed a similar pattern over the past 30 years, with mortality risk rising progressively with age. This trend was more gradual in the high SDI region, suggesting relatively better control. Regarding period effects, both the global trend and those across all five SDI regions demonstrated a relatively steady decline over time. Cohort effects also showed similar downward patterns across regions. Notably, in the high-middle and high SDI groups, cohort effect values started at relatively high levels and declined below zero over time, indicating that IS mortality has been effectively controlled in successive birth cohorts in these regions.

Figures 3D–F illustrates the APC-estimated incidence trends for IS. Incidence risk increased with age in a pattern that was consistently observed across all countries and regions. Globally and across all SDI categories, period effects were generally negative, although most regions exhibited an upward trend after 2016. In particular, the middle SDI region experienced a shift into positive period effect values, suggesting an increase in incidence. A downward cohort effect was observed across most SDI regions and globally, except for a slight increase in the low-middle SDI group, implying rising incidence risk among recent cohorts.

#### PAD

**Figure 4:**
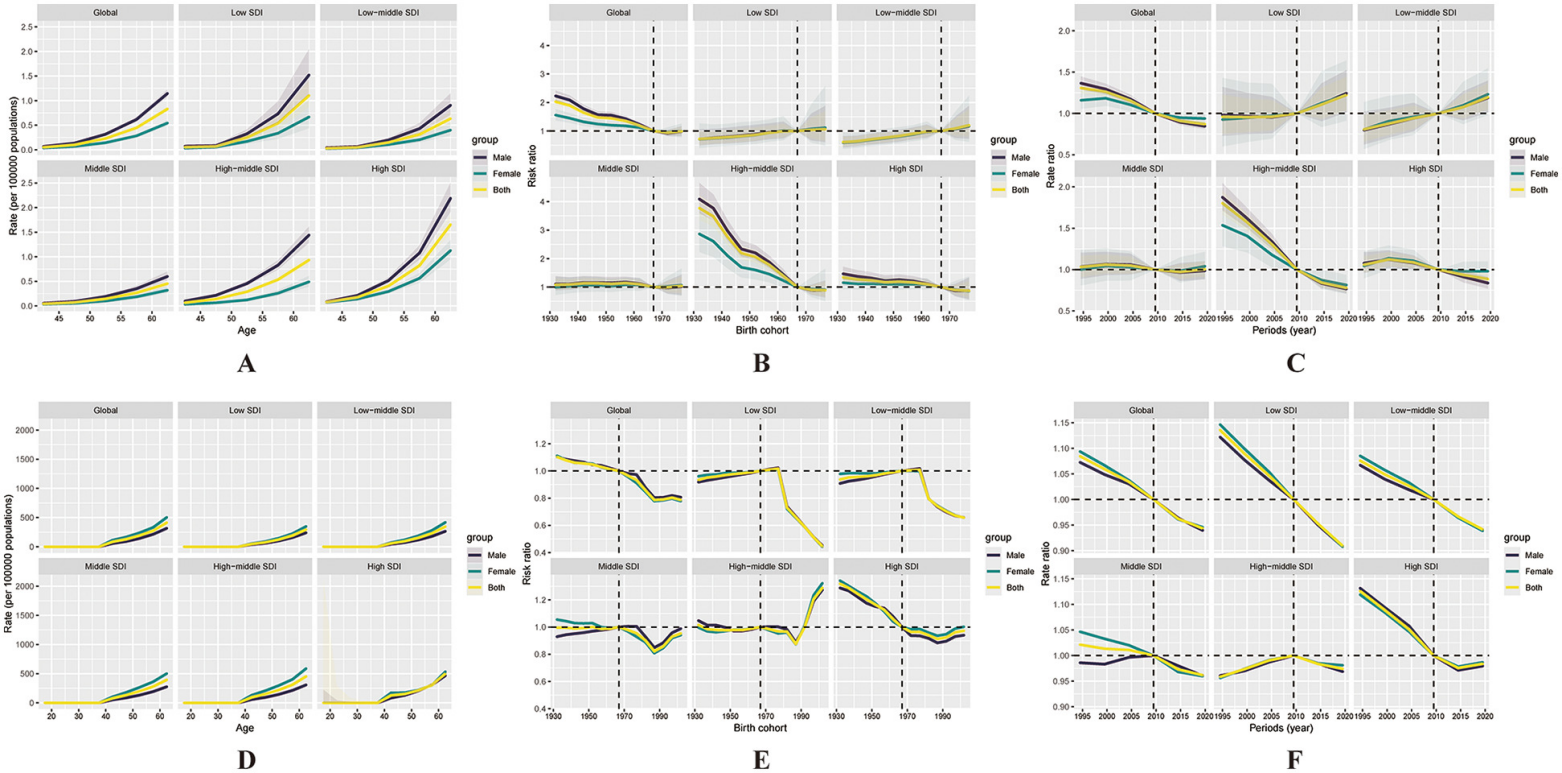
Illustrates the effects of age, period, and cohort on peripheral artery disease (PAD) mortality and incidence among the working-age population globally and across the five SDI regions. Age effects on mortality **(A)** and incidence **(D)** are presented as longitudinally fitted age-specific rates (per 100,000 person-years), adjusted for period deviations. Period effects **(B, E)** are shown as relative risks of mortality and incidence, with 2007 designated as the reference year. Cohort effects **(C, F)** are expressed as relative risks for mortality and incidence, respectively. In all panels, the dots represent point estimates of the rates or relative risks, and the shaded areas indicate the corresponding 95% confidence intervals.

Figures 4A–C shows the estimated APC effects on mortality from PAD. From 1992 to 2021, PAD mortality risk increased with age globally, with the most pronounced age-related increases observed in the high and low SDI regions, as reflected by steeper age curves. Regarding period effects, a decreasing trend in mortality risk was observed over time in the global population as well as within the high and high-middle SDI categories. Within countries classified as low or low-middle SDI, the period effect rose steadily and eventually crossed the reference level, reflecting a heightened risk in recent years. Cohort effects revealed a declining trend in mortality risk at the global level and within the high and high-middle SDI groups, but a rising trajectory was observed in low and low-middle SDI groups.

Figures 4D–F presents the estimated APC effects on the incidence of PAD. From 1992 to 2021, the incidence of PAD increased markedly with age at the global level, particularly in the high, high-middle, and middle SDI regions, where the age effect curves were steep and indicated a rapid rise in risk with age. Although the low and low-middle SDI regions followed a similar upward trajectory, the rate of increase was less pronounced. Regarding period effects, a downward trend in PAD incidence risk was observed over time in the global, high, low, and low-middle SDI regions, as period effect values declined from above 1 toward or below the reference level. While the global cohort effect showed a slight decline over time, the high-middle SDI region exhibited a reversal, with the curve exceeding RR = 1 after the 1987–1996 cohorts, implying increased PAD risk in younger generations.

## Discussion

Based on the available literature, no prior study has employed the APC model to assess ASCVD burden in the working-age population across all five SDI regions. This study has several strengths. First, it focuses on the working-age population—a key component of the labor force—whose health status plays a critical role in household stability and socio-economic development. Second, we conducted A systematic investigation of longitudinal trends in incidence and mortality rates during 1992–2021, covering trends at the global and regional levels, and provided detailed insights into the three major subtypes of ASCVD—IHD, IS, and PAD—offering a precise description of disease burden across different age groups.

Compared to previous GBD 2019-based studies (16), our study further incorporated the APC model to identify key drivers of burden trends through the decomposition of age, period, and cohort effects. This not only enhanced the interpretability of trend variations but also revealed both interregional and intraregional heterogeneity. Furthermore, we assessed local drift values between 1992 and 2021 globally and across SDI regions, enabling a comprehensive analysis of incidence and mortality trends by age group and highlighting persistent burdens in specific populations that have yet to be mitigated.

Currently, there is a lack of effective global screening and intervention strategies targeting early-stage ASCVD, particularly among working-age populations. Understanding the temporal shifts and generational transitions in ASCVD burden is crucial for informing these strategies. To achieve this, we employed the APC modeling framework using the Web Tool developed by the U.S. National Cancer Institute (15), which enables the decomposition of overall trends into age, period, and cohort components.

While APC modeling is well suited to this task, it carries the methodological challenge of non-identifiability due to the exact linear dependency among age, period, and cohort (cohort = period−age). The APC Web Tool addresses this issue through constraint-based estimable functions—such as net drift, local drift, longitudinal age curves, and period/cohort rate ratios—that allow meaningful interpretation of time-based changes while circumventing the identifiability problem.

We selected the APC model over joinpoint regression and other time-trend methods because of its strength in identifying generational risk shifts and age-standardized temporal patterns. This level of insight is particularly valuable for shaping targeted prevention efforts and informing long-term ASCVD control strategies.

Our findings indicate that IHD remains one of the leading global public health threats. Over the period 1992–2021, incident cases and mortality increased by 0.52% and 42.76%, respectively, changes that were largely attributable to demographic transitions, particularly population expansion and aging. While high and high-middle SDI regions experienced declines in IHD-related deaths, middle- and lower-SDI countries—as well as the global average—showed continued increases. However, after age standardization, mortality rates decreased across all SDI categories and globally. Notably, higher SDI regions consistently exhibited lower age-standardized mortality rates, whereas lower SDI regions maintained higher rates—an observation aligned with previous findings on global disparities in cardiovascular disease burden (17).

The observed heterogeneity in incidence patterns across SDI regions was characterized by overall declines in most categories, except for the middle SDI region, where age-standardized incidence increased. These findings suggest that beyond demographic factors, other determinants are influencing disease trends. Therefore, tailored early prevention strategies are needed by region. For instance, high-SDI regions should prioritize control of elevated LDL cholesterol, systolic blood pressure, BMI, high-fat diets, and tobacco use (18), while middle-SDI countries should focus more on fasting plasma glucose, air pollution, and smoking (19).

As the most common type of stroke, IS also presents a considerable burden. Earlier studies reported that approximately 85% of all strokes are classified as ischemic (20). The GBD 2021 study estimated that IS made up 65.3% of all new stroke cases (21), an increase from 62.4% in GBD 2019 (22). Over the past 30 years, both IS incidence and deaths have increased globally. Although age-standardized rates have declined, suggesting reductions in individual risk after adjusting for demographic changes, the absolute burden remains high—especially in SDI regions outside of the high and high-middle groups. This reinforces the ongoing socio-economic disparities in the global IS burden (23, 24). Health advantages in high-SDI countries are likely linked to stronger health systems, effective interventions, and greater public engagement (25). In contrast, lower SDI regions are confronted with persistent challenges, including limited access to healthcare services, inadequate risk factor management, and insufficient implementation of secondary prevention strategies (26). Effective IS prevention efforts should focus on the five major stroke-related risk factors: high BMI, elevated ambient temperature, high fasting glucose, consumption of sugar-sweetened beverages, and physical inactivity (21).

Compared to IHD and IS, PAD exhibits a distinct burden pattern. In high and high-middle SDI regions, incidence rates have shown a consistent decline over time, likely due to shared pathophysiological mechanisms with coronary artery disease, allowing PAD to benefit from existing cardiovascular prevention strategies (27). Unlike the higher SDI regions, the middle, low-middle, and low SDI categories exhibited increasing PAD incidence over the study period. These disparities may be driven not only by demographic transitions but also by the inadequate implementation of primary prevention measures (28, 29). In addition, public awareness of PAD remains low—only 25.8% of the population is aware of the disease, much lower than for stroke and coronary heart disease (30)—potentially leading to delayed diagnosis and treatment. It was not until 2005 that the American Heart Association (AHA) and the American College of Cardiology (ACC) recommended an ankle-brachial index (ABI) < 0.90 as a reliable diagnostic threshold for PAD, which helped to standardize diagnosis and improve comparability across studies (31).

Taken together, despite global declines in age-standardized ASCVD incidence and mortality rates, the absolute number of cases and deaths among the working-age population continues to increase—particularly in low- and middle-SDI regions—posing a growing public health challenge. Given the central social and economic roles of working-age individuals, the loss or reduction of their productivity can have far-reaching implications. Therefore, region-specific and precision-based interventions should be developed according to SDI level, with emphasis on health education, risk factor management, and early screening. Multilevel strategies at both the population and individual levels are essential to mitigate the health and socio-economic burden of ASCVD among working-age adults.

### Limitations

Despite the strengths of this study in systematically analyzing disease trends and introducing methodological innovations, several limitations should be acknowledged. First, the analysis was based on the GBD 2021 dataset, which contains data of variable quality and completeness—especially in less developed regions. Many estimates rely heavily on covariate-driven modeling, which may affect the accuracy of ASCVD burden estimates in these areas. Moreover, although this study defined the working-age population as 15–64 years, the GBD database provides PAD estimates only for individuals aged 40 years and above. As a result, PAD incidence in the 15–39 age group was assumed to be zero in the APC model due to the lack of available data. This assumption may lead to an underestimation of the true disease burden among younger adults, particularly in high-risk subpopulations such as individuals with diabetes or a history of smoking. Although continuous improvements in data processing and modeling techniques have enhanced the precision of estimates, the acquisition of high-quality primary data remains critical for improving accuracy. Second, although age-standardization was performed during data processing, uncertainty in data quality control persists due to variability in data collection procedures, coding systems, and reporting standards across countries. This may introduce bias or measurement error, potentially affecting the validity and comparability of the findings. Therefore, caution is warranted when interpreting the results. Third, the APC model typically uses five-year age group intervals to ensure model stability; however, this may limit its sensitivity to detect subtle temporal fluctuations, especially in regions undergoing rapid epidemiological transitions. Fourth, the reliability of PAD mortality estimates may be affected by coding heterogeneity in vital registration systems, especially in low-SDI regions. PAD is often underdiagnosed or miscoded on death certificates due to lack of awareness or limited diagnostic infrastructure. As GBD data are derived largely from ICD-coded sources, such underreporting may bias mortality trends and lead to underestimation of the true PAD burden. This limitation is particularly relevant when interpreting regional disparities and trends over time. Lastly, the present study did not conduct more granular analyses at the national level. Given the substantial differences in health conditions and healthcare resource distribution across countries, future research incorporating country-level data and cohort-based approaches will be valuable in accurately assessing the relative risk of ASCVD and identifying targeted interventions for the working-age population in different settings.

## Conclusion

This study underscores that although significant progress has been made in reducing the incidence and mortality of ASCVD among the working-age population at both global and SDI-region levels, the disease remains a critical public health threat. As the core labor force, working-age individuals are particularly vulnerable to ASCVD, and their health status is closely tied to socio-economic development. The findings suggest that context-specific, accessible healthcare strategies should be formulated based on the socio-economic and healthcare resource profiles of different SDI regions. Furthermore, to curb the ongoing epidemic of ASCVD, multisectoral collaboration is urgently needed to promote cross-sector public health interventions and coordinated resource integration, ultimately improving cardiovascular health among the global working-age population.

## Data Availability

The datasets presented in this study can be found in online repositories. The names of the repository/repositories and accession number(s) can be found below: https://vizhub.healthdata.org/gbd-results.
